# Factors associated with the choice of having multiple sexual partners among male college students with casual heterosexual sex in Zhejiang Province, Eastern China

**DOI:** 10.1186/s12879-023-08796-7

**Published:** 2023-11-10

**Authors:** Zhongrong Yang, Weiyong Chen, Qiaoqin Ma, Wanjun Chen, Xin Zhou, Hui Wang, Tingting Jiang

**Affiliations:** 1 Huzhou Center for Disease Control and Prevention, Huzhou, 313000 Zhejiang Province China; 2 Department of HIV/STD control and prevention, Zhejiang Provincial Center for Disease Control and Prevention, No.3399, Binsheng Road, Hangzhou, 310051 Zhejiang Province China

**Keywords:** Casual sexual behavior, HIV, AIDS, Factors, Male College students

## Abstract

**Background:**

Young students infected with HIV have become a significant public health issue in China, this study aimed to understand the factors influencing the choice of having multiple sexual partners among male college students who had casual heterosexual sex in Zhejiang Province and to scientifically justify developing HIV/AIDS intervention strategies among them.

**Methods:**

A stratified cluster sampling method was used for the survey of students from 13 colleges or universities in Zhejiang Province between October and November 2018. The questionnaire collected information on general demographic characteristics, knowledge of HIV/AIDS prevention and treatment, sexual attitudes and risk awareness, sexual behavioural characteristics, and acceptance of interventions. The univariable and multivariable analyses were conducted in this study.

**Results:**

Study participants included 362 male college students who exhibited casual heterosexual sex and were aware of the number of sexual partners they had. Among them, 222 students engaged in casual heterosexual sex with multiple sexual partners (61.33%). The results of the multivariable analysis revealed several factors associated with male students’ choice to have multiple sexual partners: monthly living expenses greater than or equal to 1501 CNY (*adjusted OR* = 2.24, 95% *CI* = 1.21–4.16), sexual behavior after consuming alcohol (*adjusted OR* = 2.19, 95% *CI* = 1.32–3.63), whose casual partner types were non-student (*adjusted OR* = 2.51, 95% *CI* = 1.45–4.22), and those who discussed using condoms during sexual intercourse (*adjusted OR* = 0.50, 95% *CI* = 0.28–0.89).

**Conclusion:**

The choice to engage in casual heterosexual sex with multiple partners was found to be associated with several factors among male college students, including economic status, engaging in sexual behavior after consuming alcohol, the type of the casual partner, and using condoms. These findings highlight the significance of implementing targeted interventions and comprehensive sexual health education programs within college settings in order to encourage safer sexual practices among students.

## Background

In recent years, the HIV/AIDS epidemic has remained a severe global public health problem [1–3]. Although highly effective antiretroviral therapy can reduce the viral load in people living with HIV, it cannot completely remove the virus from the patient’s body and requires long-term or lifetime medication [4]. HIV/AIDS has a significant impact on patients’ quality of life and mental well-being. Moreover, young students infected with HIV encounter substantial challenges in their academic life and studies [5].

Young students contracting HIV have drawn widespread social attention [6]. In recent years, the number of newly reported HIV/AIDS cases among young students in China has remained at approximately 3000 every year [7]. Sexual transmission is widely acknowledged as the primary factor contributing to HIV infection, young students are often sexually active, some male college students may exhibit strong sexual desires and curiosity, prompting them to explore a variety of sexual experiences [8, 9]. During the rapid transition from adolescence to adulthood, college students experience various physical and psychological changes. The combination of increased demand for sexual activity and a lack of awareness about self-prevention and protection has resulted in a high prevalence of HIV infection among this population [10]. Effective curbing of the spread of HIV among young students is critical for controlling the HIV/AIDS epidemic in China.

Currently, most college students living with HIV/AIDS are male [9], and the most common way for male college students to be infected with HIV is men who have sex with men (MSM) transmission. Many studies have explored homosexual behaviour characteristics and intervention measures for college students [11–13]. Despite studies linking high infection rates with homosexuality, heterosexual transmission remains the primary cause of a large majority of HIV infections. Casual heterosexual partner is also associated with a high risk of HIV transmission [14]. Therefore, research on heterosexual transmission should not be ignored.

In recent years, there has been a growing interest in the issues surrounding male college students who engage in heterosexual behavior and have multiple sexual partners [15, 16]. Limited research has been conducted on the characteristics and associated factors of male college students who engage in casual heterosexual sex and have multiple sexual partners. A high proportion of these participants also failed to use condoms consistently with casual sex partners which then involved unsafe sex and may increase the risk of HIV/STIs acquisition [17]. It is necessary to promote and popularize relevant knowledge to improve their cognitive level if male college students lack sufficient sexual health knowledge and self-protection awareness. Therefore, by analysing the situation of multiple sexual partners and associated factors among male college students engaging in casual heterosexual sex, we can establish a scientific foundation for the development of HIV/AIDS intervention strategies among this population.

## Materials and methods

### Study design

A cross-sectional survey method was conducted in this study.We surveyed students from 13 colleges and universities in Zhejiang Province between October and November 2018. A stratified cluster sampling method was adopted, and three departments were selected from each college/university using the random number table method. Subsequently, the classes were selected for each department by grade. The participants were male college students who had reported casual heterosexual sex in the past year and were aware of the number of casual sexual partners they had engaged with. The teachers informed the college students to complete online electronic questionnaires. Undergraduates who were not currently on campus would uniformly send out the questionnaire network link, and they filled in the questionnaire by themselves following the survey instructions. This study was a follow-up and further investigation on male college students who have heterosexual behaviors. We took into account the interference of students who were MSM, and participants in this group had been excluded from the analysis.

### Participants

**Fig. 1:**
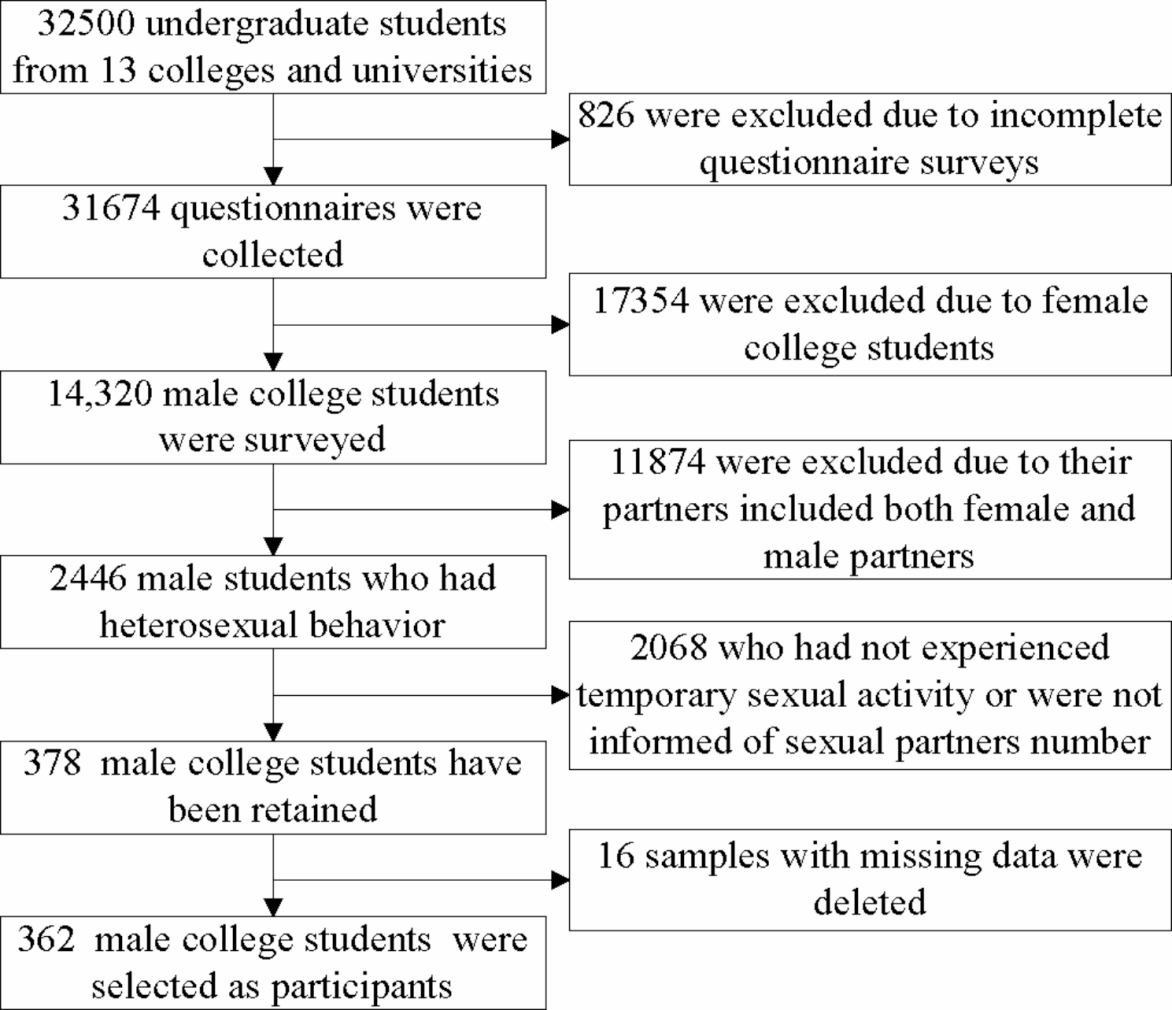
The flowchart for the inclusion and exclusion process

We recruited 32,500 undergraduate students from 13 colleges and universities and collected 31,674 questionnaires, with a response rate of 97.46%. Among them, 14,320 male college students were surveyed. 2665 (18.61%) of whomself-reported having a sexual partner whom informed their partners included both female and male partners, including 219 MSM. 16 samples with missing data were deleted, 362 (13.58%) male college students who had experienced casual sexual activity and were informed of the number of sexual partners were selected from all 2446 male students who had heterosexual behavior as the participants. The flowchart for the inclusion and exclusion process was shown in Fig. 1.

### Ethical statement

This study protocol was approved by ethics committee of the Zhejiang Provincial Center for Disease Control and Prevention, and this study complied with the declaration of Helsinki and all methods were performed in accordance with the relevant guidelines and regulations of the ethics committee of the Zhejiang Provincial Center for Disease Control and Prevention (No.2018-036). All the participants provided written informed consent.

### Contents of the questionnaire

The main contents of the questionnaire included general demographic characteristics, knowledge of HIV/AIDS prevention and treatment (included whether it can be judged by appearance that a person is infected with HIV, whether daily life and study contact will spread HIV, have you learned about HIV/AIDS through the school network in the past year, have you received any publicity about HIV testing from the school in the last year, have you received an HIV risk self-assessment conducted by the school in the past year), sexual attitudes and risk awareness (included will you accept one night stand, do you accept commercial sex, whether discussed using condoms during sexual intercourse, do you want to know that your sex partner may be infected with HIV, do you think that you are at risk of contracting HIV), sexual behavioural characteristics (included casual partner types, condom use with casual partners, have you had sex with a fixed partner in the past year, have you ever had sexual behavior after consuming alcohol, have there ever been commercial sex acts involving money transactions, how about condom use during sex with a regular partner in the past year), and acceptance of interventions (included have you received voluntary counseling and testing).

### Definition of related indicators

Casual heterosexual behavior: it means casual sexual behavior who engaged in sex with a partner of the opposite sex. Heterosexual casual multiple partner behaviour: this refers to casual heterosexual sex in the past year which occurred occasional sexual activity with two or more heterosexual partners. Casual sexual partner types: According to the source of casual partners in the previous year, the two categories were divided into school students and non-students. Monthly living expenses: refers to how much money the participant spends per month.

### Statistical analysis

SPSS software (version 21.0; SPSS Inc., Chicago, IL, USA) was used for data analysis. Variables such as age, grade, and sexual behaviour characteristics were expressed as means, composition ratios, or rates. The chi-square test was used to compare the demographic characteristics of male college students with casual heterosexual sex. The dependent variable was defined as participants who self-reported casual heterosexual sex and were aware of the number of sexual partners they had engaged with. The independent variables included demographic characteristics, knowledge of HIV/AIDS prevention, sexual attitudes, characteristics of sexual behaviour, and acceptance of HIV/AIDS interventions. The single-factor logistic regression method of univariable analysis was used to analyse the associated factors of multiple sexual partners among participants; variables with *P* < 0.20 in the univariable analysis, and demographic characteristics were included as independent variables in the multivariable logistic regression analysis [18]. Differences were considered statistically significant at *P* < 0.05.

## Results

### General demographic characteristics

A total of 362 participants aged between 18 and 28 years old, with an average age of 20.08 ± 1.37 participated in the study. Among them, 222 male college students (61.33%) had casual heterosexual sex with multiple sexual partners, and the average age was 20.05 ± 1.40. There were no statistical differences between multiple sexual partners among participants in terms of age, grade, household registration, or family relationship (Table 1). 46.8% of respondents in “multiple sexual partners” group were town/city, and 35.7% of participants in the comparable group were town/city, the chi-square analysis indicated that there was significant difference (χ^2^ = 4.353, *P* < 0.05) between two groups. 49.5% of respondents in “multiple sexual partners” group’s monthly living expenses were greater than or equal to 1501, and 30.7% of participants in the comparable group were greater than or equal to 1501, the chi-square analysis indicated that there was significant difference (χ^2^ = 12.555, *P* < 0.01) between two groups.

**Table 1:** Demographic characteristics of participants

Variables	Multiple sexual partners group (*n* = 222, %)		Single partner group (n = 140, %)			χ^2^		*P*
**Age (yrs)**	20.05 ± 1.404		20.11 ± 1.331					
Less than or equal to 19	80(36.0)		46(32.9)			0.391		0.822
20–21	108(48.6)		71(50.7)					
Greater than or equal to 22	34(15.4)		23(16.4)					
**Grade**								
Freshman	52(23.4)		28(20.0)			1.609		0.657
Sophomore	70(31.5)		50(35.7)					
Junior	75(33.8)		50(35.7)					
Senior	25(11.3)		12(8.6)					
**Province of household registration**						2.672		0.102
Other provinces	70(31.5)		33(23.6)					
Zhejiang Province	152(68.5)		107(76.4)					
**Source of origin hometown**						4.353		0.037
Rural area	118(53.2)		90(64.3)					
Town/city	104(46.8)		50(35.7)					
Monthly living expenses (CNY*)							12.555	0.002
Less than or equal to 1000	54(24.3)		45(32.1)					
1001–1500	58(26.1)		52(37.2)					
Greater than or equal to 1501	110(49.6)		43(30.7)					
**Family relations**						0.139		0.710
Harmonious	177(79.7)		109(77.9)					
General/disharmonious	45(20.3)		31(22.1)					

* CNY, Chinese Yuan

### Analysis of the factors influencing the choice of having multiple sexual partners among participants

In the univariable analysis (Table 2), participants who had accepted one-night stands (*crude OR* = 2.13, 95% *CI* = 1.26–3.63), those who had accepted commercial sex (*crude OR* = 2.23, 95% *CI* = 1.45–3.44), those whose casual partners were non-student (*crude OR* = 2.83, 95% *CI* = 1.75–4.57), and those who engaged in casual sexual behaviours after consuming alcohol (*crude OR* = 2.47, 95% *CI* = 1.58–3.85) were more likely to have multiple sexual partners. Those who discussed condom use during sexual intercourse (*crude OR* = 0.50, 95% *CI* = 0.30–0.83) were less likely to have multiple sexual partners.

**Table 2:** Influencing factors analysis of multiple sexual partners among 362 participants

Variables	Multiple sexual partners group (n = 222)		Single partner group (n = 140)		Univariable analysis				Multivariable analysis		
	*n* (%)		*n* (%)		*OR*(95%*CI*)		*P*		a*OR*(95%*CI*)	*P*	
**Whether it can be judged by appearance that a person is infected with HIV?**											
Wrong/don’t know	46(20.7)		27(19.3)		Ref				——	——	
Correct	176(79.3)		113(80.7)		0.91(0.54–1.55)		0.740		——	——	
**Whether daily life and study contact will spread HIV?**											
Wrong/don’t know	42(18.9)		31(22.1)		Ref				——	——	
Correct	180(81.1)		109(77.9)		1.22(0.72–2.05)		0.475		——	——	
**Have you learned about HIV/AIDS through the school network in the past year?**											
No	164(73.9)		107(76.4)		Ref				——	——	
Yes	58(26.1)		33(23.6)		1.15(0.70–1.88)		0.585		——	——	
**Have you received any publicity about HIV testing from the school in the last year?**											
No	74(33.3)		41(29.3)		Ref				——	——	
Yes	148(66.7)		99(70.7)		0.83(0.52–1.31)		0.421		——	——	
**Have you received an HIV risk self-assessment conducted by the school in the past year?**											
No	115(51.8)		69(49.3)			Ref			——	——	
Yes	107(48.2)		71(50.7)			0.90(0.59–1.38)	0.641		——	——	
**Age (yrs)**											
Less than or equal to 19	80(36.0)		46(32.9)			Ref			Ref		
20–21	108(48.7)		71(50.7)			0.88(0.55–1.40)	0.577		0.82(0.48–1.40)	0.460	
Greater than or equal to 22	34(15.3)		23(16.4)			0.85(0.45–1.61)	0.620		0.71(0.35–1.45)	0.346	
**Province of household registration**											
Other provinces	70(31.5)		33(23.6)		Ref				Ref		
Zhejiang Province	152(68.5)		107(76.4)		0.67(0.41–1.09)		0.103		0.65(0.38–1.12)	0.121	
**Source of origin hometown**											
Rural area	118(53.2)		90(64.3)		Ref				Ref		
Town/city	104(46.8)		50(35.7)		1.59(1.03–2.45)		0.037		1.39(0.84–2.30)	0.201	
Monthly living expenses (CNY*)											
Less than or equal to 1000	54(24.3)		45(32.1)		Ref				Ref		
1001–1500	58(26.1)		52(37.2)		0.93(0.54–1.60)		0.792		1.19(0.65–2.19)	0.580	
Greater than or equal to 1501	110(49.6)		43(30.7)		2.13(1.26–3.62)		0.005		2.24(1.21–4.16)	0.010	
**Will you accept one night stand?**											
Don’t accept/don’t know	32(14.4)		37(26.4)		Ref				Ref		
Yes	190(85.6)		103(73.6)		2.13(1.26–3.63)		0.005		1.53(0.79–2.95)	0.206	
**Do you accept commercial sex?**											
Don’t accept/don’t know	83(37.4)		80(57.1)		Ref				Ref		
Yes	139(62.6)		60(42.9)		2.23(1.45–3.44)		< 0.001		1.61(0.95–2.75)	0.078	
**Have you had sex with a fixed partner in the past year?**											
No	71(32.0)		48(34.3)		Ref				——	——	
Yes	151(68.0)		92(65.7)		1.11(0.71–1.74)		0.650		——	——	
**Have you ever had sexual behavior after consuming alcohol?**											
No	106(47.7)		97(69.3)		Ref				Ref		
Yes	116(52.3)		43(30.7)		2.47(1.58–3.85)		< 0.001		2.19(1.32–3.63)	0.002	
**Whether discussed using condoms during sexual intercourse?**											
No	70(31.5)		26(18.6)		Ref				Ref		
Yes	152(68.5)		114(81.4)		0.50(0.30–0.83)		0.007		0.50(0.28–0.89)	0.018	
**Casual partner types**											
School student	123(55.4)		109(77.9)		Ref				Ref		
Non-student	99(44.6)		31(22.1)		2.83(1.75–4.57)		< 0.001		2.51(1.45–4.22)	0.001	
**Condom use with casual partners**											
Never used	32(14.4)		23(16.4)		Ref				Ref		
Sometimes/frequently used	109(49.1)		51(36.4)		1.54(0.82–2.89)		0.182		1.89(0.92–3.87)	0.082	
Every time used	81(36.5)		66(47.2)		0.88(0.47–1.65)		0.695		1.04(0.50–2.16)	0.917	
**Have there ever been commercial sex acts involving money transactions?**											
No	163(73.4)		108(77.1)		Ref				——	——	
Yes	59(26.6)		32(22.9)		1.22(0.75-2.00)		0.472		——	——	
**Do you want to know that your sex partner may be infected with HIV?**											
No	109(49.1)		72(51.4)		Ref				——	——	
Yes	113(50.9)		68(48.6)		1.10(0.72–1.68)		0.666		——	——	
**Do you think that you are at risk of contracting HIV?**											
No/Don’t know	199(89.6)		123(87.9)		Ref				——	——	
Yes	23(10.4)		17(12.1)		0.84(0.43–1.63)		0.599		——	——	
**Have you received voluntary counseling and testing?**											
No	206(92.8)		128(91.4)		Ref				——	——	
Yes	16(7.2)		12(8.6)		0.83(0.38–1.81)		0.636		——	——	
**How about condom use during sex with a regular partner in the past year? (n = 243)**											
Never used	22(14.6)		15(16.3)		Ref				——	——	
Sometimes/frequently used	78(51.6)		38(41.3)		1.40(0.65-3.00)		0.387		——	——	
Every time used	51(33.8)		39(42.4)		0.89(0.41–1.94)		0.772		——	——	

^*^ CNY, Chinese Yuan

In the multivariable analysis (Table 2), there were no statistical differences in age, province of household registration, source of origin hometown, acceptance of a one-night stand, acceptance of commercial sex, or condom use with casual partners among participants (*P* > 0.05). The results showed that participants were more likely to have multiple sexual partners if their monthly living expenses were greater than or equal to 1501 CNY (*adjusted OR* = 2.24, 95% *CI* = 1.21–4.16), had sexual behavior after consuming alcohol (*adjusted OR* = 2.19, 95% *CI* = 1.32–3.63), and had casual sexual partners who were non-student (*adjusted OR* = 2.51, 95% *CI* = 1.45–4.22). Participants who discussed using condoms during sexual intercourse (*adjusted OR* = 0.50, 95% *CI* = 0.28–0.89) were less likely to have multiple sexual partners.

## Discussion

This research conducted a cross-sectional survey of college students in Zhejiang Province and analysed the current situation and associated factors of multiple sexual partners among male college students engaged in casual heterosexual sex. The results showed that 13.58% of male college students engaged in casual heterosexual sex and were aware of the number of their sexual partners they had engaged with. Among them, 61.33% students with casual heterosexual sex had multiple sexual partners. The survey indicated that male college students with casual heterosexual sex were more likely to have multiple sexual partners if they showed the following characteristics: monthly living expenses were greater than or equal to 1501 CNY; engaged in sexual behavior after consuming alcohol; and those who had casual sexual partners who were non-students. In contrast, those who discussed using condoms during sexual intercourse were less likely to have multiple sexual partners, compared to the comparison group. This study offers valuable insights into the current situation and associated factors of multiple sexual partners among male college students engaged in casual heterosexual behavior. The findings contribute to the field of public health, emphasizing the need for targeted interventions, education programs, and policies addressing the specific challenges faced by this population. Public health professionals can promote healthy sexual behaviors and reduce the risks associated with multiple sexual partners among male college students in Zhejiang Province and beyond by addressing these factors.

This study suggested that the economic status of young students may be one of the factors influencing engagement with multiple sexual partners among college students who engaged in casual heterosexual sex. Male college students with higher monthly living expenses are more likely to engage in sexual behaviors with multiple partners than those with lower expenses [19]. This may be due to the fact that these students have more disposable income, which allows them to engage in activities that require spending money and to meet multiple potential sexual partners. Therefore, personalized HIV/AIDS interventions need to implement for young students urgently. Timely and appropriate sex education can enable college students to acquire scientific knowledge of sexual health and HIV/AIDS and cultivate correct sexual concepts and healthy sexual behaviours [20].

The findings of this research indicated that alcohol consumption may be one of the factors affecting multiple sexual partners among participants. While alcohol consumption has the potential to impair judgment and decision-making, it can also lead to the adoption of unsafe sexual behaviors. It is crucial to acknowledge that individuals who are intoxicated might have an increased susceptibility to engaging in unprotected sexual activities, thereby elevating their vulnerability to contracting HIV. Previous studies have shown that male college students are more likely have high risk sexual behaviors than female college students, such as one-night stands, multiple sexual partners, and not using condoms after consuming alcohol [21–23].Nearly two-thirds of American college students’ one-night stands are related to consuming alcohol behaviour [24, 25]. HIV/AIDS prevention can be combined with moderating consuming alcohol behaviour for behavioural interventions among male college students [26].It is recommended that universities and colleges provide education on responsible consuming alcohol and safe sexual practices to reduce the risk of negative outcomes associated with alcohol use and multiple sexual partners. This includes promoting responsible alcohol use, encouraging the use of condoms, and providing help for students who may be struggling with problematic consuming alcohol or sexual behaviors.

This survey showed that participants whose casual partner types were non-students might be one of the factors that affect the emergence of multiple sexual partners. Owing to the complex social interactions among non-students outside campus, male college students may face a high risk of sexually transmitted infections when interacting with them. Therefore, comprehensive intervention measures are needed to reduce the behaviour of engaging with multiple sexual partners among male college students. For example, these measures include providing a good learning environment for male college students, improving their own psychological quality, improving their ability to make friends, responding to the pressure of social intercourse from partners, increasing the awareness of participants to take the initiative in HIV testing [11], and providing them with health education for HIV/AIDS prevention.

The study findings revealed that individuals who openly discussed using condom during sexual intercourse were less likely to engage in multiple sexual partners. Thus, a certain understanding of the protective effect of condoms’ may be one of the factors that influence the appearance of multiple sexual partners for male college students who engage in casual heterosexual sex. Studies have shown that consistent use of condoms is an effective means of preventing HIV transmission [27–29].Condom use is an important method of reducing the risk of sexually transmitted infections and unintended pregnancies, and it is recommended that all sexually active individuals use condoms consistently and correctly. However, some male college students may be reluctant to use condoms due to various reasons, such as discomfort or reduced sensitivity during sex. It is recommended that universities and colleges provide education on safe sex practices, including the proper use of condoms and alternatives to traditional condoms, such as female condoms. In addition, it is important to address any concerns or misconceptions that students may have about condom use to promote greater uptake and consistent use.

This study has some limitations. First, this questionnaire was not designed specifically for male college students who had casual heterosexual sex, and the content of the survey was self-reported by participants, who may have recall bias. Owing to personal privacy, participants may not be entirely honest about their own sexual history or their sexual partners. Therefore, there may also be an information bias. However, this study performed multivariable logistic regression analysis to reduce the influence of confounding factors. In addition, because this was a cross-sectional study, only the current status of the participants could be determined through a questionnaire survey, and no further prospective cohort follow-up studies had been conducted; therefore, there were no causal inferences in this study. Meanwhile, the lack of data on the economic family status of students, age at initial sexual debut, and assessment qualitative assessment of participants’ choice to engage in sexual activities with multiple casual partners are significant limitations that affect the generalization of this study and should be noted. In the future, multicentre prospective cohort follow-up observations involving more research variables should be conducted to verify the results of this study.

In conclusion, the phenomenon of engaging with multiple sexual partners among male college students with casual heterosexual sex is common, and there may be a potential risk of HIV transmission. Effective HIV/AIDS intervention strategies should be developed to prevent unsafe sex among male college students. Overall, the findings from this research provide valuable insights for public health practitioners, policymakers, and researchers, highlighting the importance of targeted interventions and comprehensive sex education in promoting safe sexual behaviors among college students.

## Data Availability

The datasets used and/or analyzed during the current study are available from the corresponding author upon reasonable request.

## Abbreviations

95% *CI*: 95% confidence interval
HIV: Human immunodeficiency virus
AIDS: Acquired immunodeficiency syndrome
CNY: Chinese Yuan
HAART: Highly effective antiretroviral therapy
